# Cancer risk in Vietnam war veterans from the Korean Vietnam war veterans’ health study cohort

**DOI:** 10.3389/fonc.2023.1048820

**Published:** 2023-01-25

**Authors:** Wanhyung Lee, Soyoung Park, Seong-Kyu Kang, Seunghon Ham, Jin-Ha Yoon, Won-Jun Choi

**Affiliations:** ^1^ Department of Occupational and Environmental Medicine, Gil Medical Center, Gachon University College of Medicine, Incheon, Republic of Korea; ^2^ Department of Occupational and Environmental Medicine, Kangbuk Samsung Hospital, Sungkyunkwan University School of Medicine, Seoul, Republic of Korea; ^3^ Department of Preventive Medicine, Yonsei University College of Medicine, Seoul, Republic of Korea; ^4^ The Institute for Occupational Health, Yonsei University College of Medicine, Seoul, Republic of Korea

**Keywords:** KOVECO, agent orange, vietnam war, veterans, cancer

## Abstract

**Introduction:**

During the Vietnam War, several unknown chemicals, such as Agent Orange, were used in Vietnam by the military. Therefore, there have been continuous health concerns among the Vietnamese population and veterans exposed to these hazardous chemicals. This study aimed to investigate the risk of all cancers and also organ-specific cancers among Korean veterans of the Vietnam War.

**Methods:**

This study used a national representative cohort that included all Korean Vietnam War veterans as the interest group, with 1:4 age-sex-region-matched general Korean citizens as the reference group, from 2002 to 2018. Age-standardized incidence ratios (SIRs) and 95% confidence intervals (CIs) were calculated for all cancers and for 31 organ-specific cancer categories based on the medical facility visit data.

**Results:**

An increased SIR of 1.07 (95% CI, 1.06–1.08) was observed for all cancers among the veterans. There was a significantly increased risk of cancer among 22/31 organspecific cancers, with 18 cancer categories showing a significantly higher risk than all cancers. The highest risk was observed for “malignant neoplasms of other parts of the central nervous system” (SIR, 1.71; 95% CI, 1.51–1.92).

**Discussion:**

This study evaluated the risk of cancer among Korean Vietnam War veterans. Further studies are warranted to investigate various health determinants in the veterans as well as the Vietnamese population.

## 1 Introduction

The Republic of Korea dispatched over 300,000 troops during the Vietnam War (VW) (1). There is an ongoing tragedy affecting the health of Korean VW veterans because of the various unknown chemicals used in the war. For example, VW veterans may have been exposed to defoliants, or Agent Orange (AO), during the 1960s and the early 1970s (2). AO is a herbicide that mainly comprises the phenoxy herbicides 2,4-dichlorophenoxyacetic acid and 2,4,5-trichlorophenoxyacetic acid (2,4,5-T) (3). Although various kinds of herbicides were sprayed during the VW, AO represents all the phenoxy herbicides used then. As 2,4,5-T was contaminated with dioxin as a byproduct of the manufacturing process, exposure to AO resulted in exposure to dioxin or 2,3,7,8-tetrachlorodibenzo-p-dioxin (TCDD).

There have been concerns regarding the adverse health effects from the various unknown chemicals used in the war, including cancer. The association between dioxin and some cancer types, such as soft tissue sarcoma, lymphomas, respiratory tract cancer, and prostate cancer, has been reported previously; however, there are limited or inconsistent results for some other cancer types. In Korea, Yi et al. reported cancer incidence among Korean VW veterans (4). From 1992 to 2003, 180,251 veterans were followed-up for cancer incidence. Compared to the low AO exposure group, the high AO exposure group showed significantly higher hazard ratios for cancers such as oral cancer, salivary gland cancer, stomach cancer, small intestine cancer, and lung cancer. There have been no further reports on cancer incidence in Korea since this study. Furthermore, previous studies on the cancer risk among the VW veterans focused only on veterans (5, 6).

This study aimed to investigate and compare the risk for, and status of, cancer incidence between Korean VW veterans and Korean citizens using the newly opened national representative cohort data of the Korean Vietnam War Veterans’ Health Study Cohort (KOVECO) (7).

## 2 Methods

### 2.1 Data and participants

KOVECO is a merged dataset of data from the National Health Insurance Sharing Service (NHISS, https://nhiss.nhis.or.kr/bd/ab/bdaba000eng.do) and the Ministry of Patriots and Veterans Affairs (MPVA, https://www.mpva.go.kr/english/index.do) of Korea. NHISS collects, stores, and manages data obtained from mandatory public health insurance services for all Korean citizens, which has been available to researchers for policy and academic research purposes since 2002. The NHISS database comprises data regarding hospital facility visits based on the standardized protocol of the Korea Classification of Diseases and Causes of Death, 4th edition, which corresponds to the International Classification of Diseases (ICD), 10th revision. All diagnoses were described based on ICD codes, with individual codes, dates, and regions (8). Since 1995, the MPVA of Korea has been rewarding veterans by providing them with medical services and welfare. The MPVA data include a list of veterans, identification information, and war participation information (rank, affiliation, class, date of entry into the war, and date of retirement). Thus, the KOVECO is an age-sex-region matched cohort that comprises tailored data from the MPVA, which includes information about Korean VW veterans, and the NHISS, which includes medical facility visit histories from 2002 to 2018.

**Figure 1:**
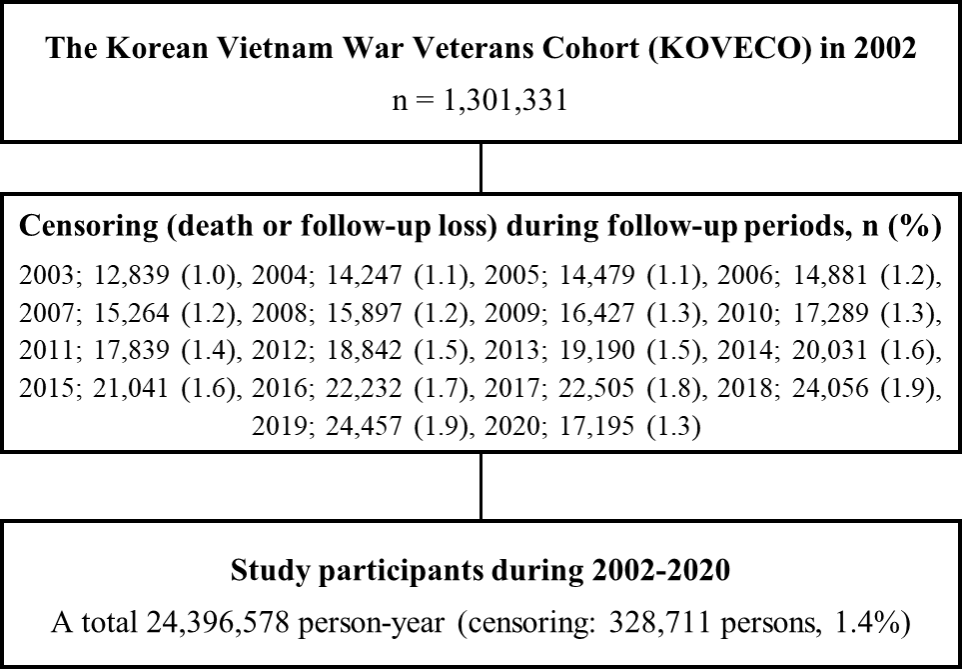
Detailed flow diagram of the study participants.

All data merging and establishing processes were conducted by an independent data specialist from the NHISS, with individual information being anonymized. A total of 1,301,331 Koreans were enrolled in KOVECO in 2002, with 250,842 VW veterans as the interest group and 1,050,489 non-veterans as the reference group. In addition, 24,396,578 person-years were observed during the follow-up period. **Figure 1** shows the detailed flow diagram of the study participants.

This study was performed in accordance with the ethical standards of the Declaration of Helsinki (1964) and its subsequent amendments. Data from the NHISS were collected after obtaining written informed consent from all the participants, and the information was anonymized. This study was approved by the Institutional Review Board of the Gachon University Gil Medical Center (IRB No. GCIRB2019-076).

### 2.2 Measurement and variables

The VW veteran group was defined based on VW-related information from the MPVA. The reference group was matched with the VW veterans group based on the 5-year-interval age groups, sex, and residence in 16 metropolitan cities in Korea in a 1:4 ratio.

Cancers were defined based on ICD diagnosis codes for malignant neoplasms for all cancers (C00–C97) and organ-specific categories according to the human organ system (9). During the follow-up period, only the first cancer diagnosis for each individual was included. The 31 organ-specific categories and their ICD codes are as follows: malignant neoplasms of lip, oral cavity, and pharynx (C00–C14); malignant neoplasms of esophagus (C15); malignant neoplasms of stomach (C16); malignant neoplasms of colon (C18); malignant neoplasms of rectosigmoid junction, rectum, anus, and anal canal (C19–C21); malignant neoplasms of liver and intrahepatic bile ducts (C22); malignant neoplasms of pancreas (C25); other malignant neoplasms of digestive organs (C17, C23, C24, C26); malignant neoplasms of larynx (C32); malignant neoplasms of trachea, bronchus, and lung (C33, C34); other malignant neoplasms of respiratory and intrathoracic organs (C30, C31, C37–C39); malignant neoplasms of bone and articular cartilage (C40, C41); malignant melanomas of skin (C43); other malignant neoplasms of skin (C44); malignant neoplasms of mesothelial and soft tissue (C45–C49); malignant neoplasms of breast (C50); malignant neoplasms of cervix uteri (C53); malignant neoplasms of other and unspecified parts of uterus (C54, C55); other malignant neoplasms of female genital organs (C51, C52, C56–C58); malignant neoplasms of prostate (C61); other malignant neoplasms of male genital organs (C60, C62, C63); malignant neoplasms of bladder (C67); other malignant neoplasms of urinary tract (C64–C66, C68); malignant neoplasms of eye and adnexa (C69); malignant neoplasms of brain (C71); malignant neoplasms of other parts of central nervous system (C70, C72); malignant neoplasms of other, ill-defined, secondary, unspecified, and multiple sites (C73–C80, C97); Hodgkin disease (C81); non-Hodgkin lymphoma (C82–C86); leukemia (C91–C95); and other malignant neoplasms of lymphoid, hematopoietic, and related tissue (C88–C90, C96).

### 2.3 Statistical analysis

We estimated the number of cases of each type of cancer during the follow-up period, based on the history of the first visit. Age-standardized incidence ratios (SIRs) and 95% confidence intervals (CIs) were calculated based on the 5-year-interval age groups. SIR was estimated using age-specific cancer incidence rates and the number of person-years in each age group of the reference group (non-VW veterans), indicating the age-specific expected incidence among VW veterans. The sum of the expected cases was compared with the total observed cases of the VW veterans, and the ratio was calculated. If the SIR and the lower limit of the 95% CI were both >1, the cancer risk was considered significantly higher in the VW veterans’ group than in the reference group. The 95% CIs were estimated using Poisson distribution. All analyses were performed using SAS, version 9.4 (SAS Institute, Cary, NC, USA).

## 3 Results

KOVECO comprised 250,842 (19.2%) VW veterans (**Table 1**). Most participants were male (99.8%), and approximately 28% had one or more neoplasms. Veterans had a significantly higher cumulative incidence of cancer than non-veterans (29.4% vs. 27.3%; p < 0.0001).

**Table 1:** Descriptive characteristics of the study participants of the Vietnam War veterans from the Korean Vietnam War Veterans’ Health Study Cohort at baseline in 2002.

	Total participants		Vietnam War veterans n (%)				p-value
	n (%)		Yes		No		
Total participants	1,301,331	(100.0)	250,842	(19.2)	1,050,489	(80.6)	
Sex							<0.0001
Male	1,299,283	(99.8)	250,489	(19.3)	1,048,794	(80.7)	
Female	2,048	(0.2)	353	(17.2)	1,695	(82.8)	
All neoplasms							<0.0001
No	940,019	(72.2)	176,928	(18.8)	763,091	(81.2)	
Yes	361,312	(27.8)	73,914	(20.5)	287,398	(79.5)	

**Figure 2:**
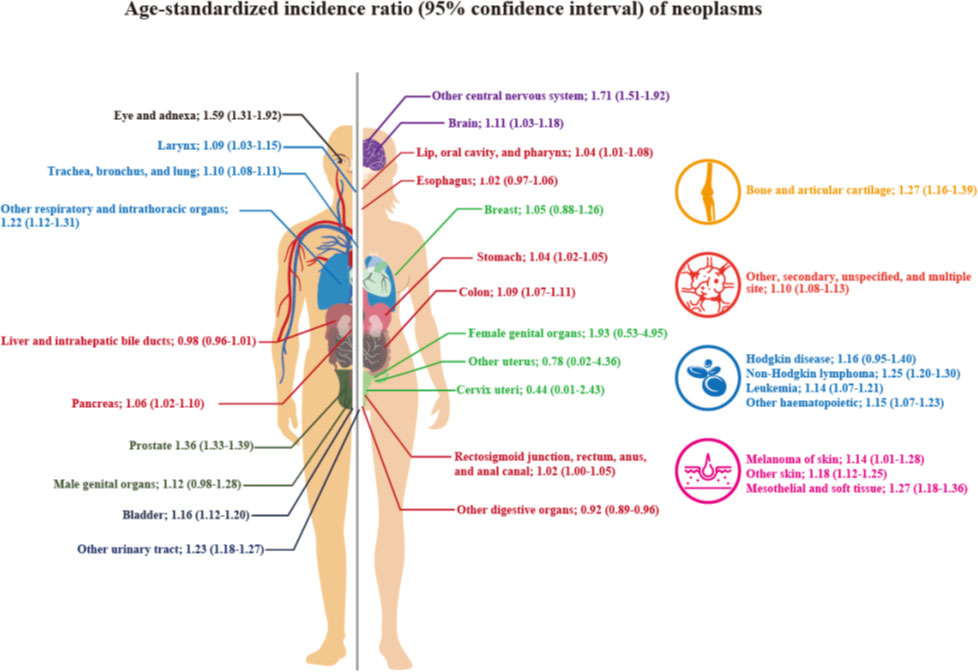
SIRs and 95% CIs according to the sites of the neoplasms.

The SIRs and 95% CIs according to neoplasm sites are shown in **Table 2** and **Figure 2**. The SIR (95% CI) for all cancer types combined, that is, all neoplasms, was 1.07 (1.06–1.08), and it was statistically significant. The SIRs (95% CIs) of the malignant neoplasms of the following organ categories were statistically significantly elevated: lip, oral cavity, and pharynx (1.04; 1.01–1.08); stomach (1.04; 1.02–1.05); colon (1.09; 1.07–1.11); pancreas (1.06; 1.02–1.10); larynx (1.09; 1.03–1.15); trachea, bronchus, and lungs (1.10; 1.08–1.11); other respiratory and intrathoracic organs (1.22; 1.12–1.31); bone and articular cartilage (1.27; 1.16–1.39); malignant melanomas of skin (1.14; 1.01–1.28); other malignant neoplasms of skin (1.18; 1.12–1.25); mesothelial and soft tissue (1.27; 1.18–1.36); prostate (1.36; 1.33–1.39); urinary bladder (1.16; 1.12–1.20); other malignant neoplasms of urinary tract (1.23; 1.18–1.27); eye and adnexa (1.59; 1.31–1.92); brain (1.11; 1.03–1.18); other parts of central nervous system (1.71; 1.51–1.92); other, ill-defined, secondary, unspecified, and multiple sites (1.10; 1.08–1.13); non–Hodgkin lymphoma (1.25; 1.20–1.30); leukemia (1.14; 1.07–1.21); and other lymphoid, hematopoietic, and related tissue (1.15; 1.07–1.23). However, the SIRs (95% CIs) of the neoplasms of the following categories were not significantly elevated: esophagus, rectum and anus, breast, other female genital organs, other male genital organs, and Hodgkin’s disease. The age-specific hospital admission status for neoplasms during the study period can be found in the **Supplementary table**.

**Table 2:** Cases and incidence rate of neoplasms among Vietnam War veterans according to cancer site.

Type of neoplasms	ICD-10a	Cases	Incidence rate per 100,000 person-year	Minimum	Maximum
All cancers	C00–C97	73,914	2,976.8	2,957.1	2,983.2
Malignant neoplasms of lip, oral cavity and pharynx	C00–C14	3,314	135.6	131.0	1,402.7
Malignant neoplasms of esophagus	C15	1,879	77.7	74.3	81.3
Malignant neoplasms of stomach	C16	16,725	684.5	674.2	695.0
Malignant neoplasms of colon	C18	8,529	352.7	345.3	360.3
Malignant neoplasms of rectosigmoid junction, rectum, anus, and anal canal	C19–C21	6,049	250.0	243.7	256.4
Malignant neoplasms of liver and intrahepatic bile ducts	C22	8,372	348.1	340.7	355.6
Malignant neoplasms of pancreas	C25	2,716	112.3	108.1	116.6
Other malignant neoplasms of digestive organs	C17, C23–C24, C26	2,856	117.8	113.4	122.1
Malignant neoplasms of larynx	C32	1,386	57.4	54.4	60.5
Malignant neoplasms of trachea, bronchus and lung	C33–C34	13,733	559.7	550.4	569.1
Other malignant neoplasms of respiratory and intrathoracic organs	C30–C31, C37–C39	646	26.9	24.9	29.1
Malignant neoplasms of bone and articular cartilage	C40–C41	458	18.9	17.2	20.7
Malignant melanomas of skin	C43	302	12.5	11.1	13.9
Other malignant neoplasms of skin	C44	1,447	59.4	56.3	62.5
Malignant neoplasms of mesothelial and soft tissue	C45–C49	788	32.7	30.4	35.0
Malignant neoplasms of breast	C50	123	5.1	4.2	6.0
Malignant neoplasms of prostate	C61	9,668	400.4	392.5	408.5
Other malignant neoplasms of male genital organs	C60, C62–C63	212	8.7	7.6	9.9
Malignant neoplasms of bladder	C67	3,364	137.9	133.2	142.6
Other malignant neoplasms of urinary tract	C64–C66, C68	2,792	115.7	111.5	120.1

Malignant neoplasms of cervix uteri (C53), Malignant neoplasms of other and unspecified parts of uterus (C54–C55), and Other malignant neoplasms of female genital organs (C51–C52, C56–C58) were excluded from the table because these cancers had less than five cases during follow-up periods.

## 4 Discussion

This study found an increased risk of all cancers among Korean VW veterans as compared to the general population. There was a significantly increased risk of cancer in 22/31 organ-specific categories, of which 18 categories showed a significantly higher risk than that of all cancers. Although the highest risk was observed for “other malignant neoplasms of the female genital organs” among VW veterans, the difference was not statistically significant. VW veterans showed a significantly increased risk for “malignant neoplasms of other parts of central nervous system.”

TCDD is a known human carcinogen (10, 11). Many experimental animal studies have provided sufficient evidence regarding its carcinogenicity. In human epidemiological studies, exposure to TCDD showed an increased risk of overall cancer related mortality, but the cancer incidence was not significantly high (12). Although the exact mechanism of carcinogenicity is not fully understood, activation of the aryl hydrocarbon (Ah) receptor is considered to be one of the most critical steps (13, 14). When TCDD binds to the Ah receptor, various biological responses occur, including gene expression, transcription, and modulation of biochemical and cellular responses (15–17). However, the carcinogenicity of TCDD in humans is debated. One of the main issues is the long biological half-life of TCDD (7–10 years) (18). The slow rate of its metabolism, and formation of epoxides, may not lead to significant damage to intracellular organelles. Moreover, Ah receptor signaling pathways are complicated, and many genes are involved in the modulation of TCDD (19, 20). These complex mechanisms may explain, at least in part, why several types of cancers, but not all, are associated with TCDD exposure. Furthermore, the peak cancer risk related to AO manifests approximately 20 years after exposure; however, the toxic effects do not disappear after that time (3).

In this study, the incidence of several cancers was significantly higher in the VW veterans than in the non-veterans. According to reviews conducted by the National Academies of Sciences, Engineering, and Medicine (3), there is sufficient evidence of an association between exposure to herbicides and soft-tissue sarcoma, non-Hodgkin lymphoma, and Hodgkin disease. However, there is limited evidence of an association between herbicide exposure and certain other cancers, such as laryngeal cancer, cancer of the lungs, bronchi, or trachea, prostate cancer, and urinary bladder cancer. Although a previous Korean veterans study also indicated an increased risk of specific cancers, only prostate cancer showed a significantly increased risk due to limited observational periods and small number of cases of cancers (21). The current study demonstrated an elevated number of significantly increased risks of specific cancers, and most results of this study seem to corroborate previous reports (3). There were some notable results in our study.

We found a significantly higher incidence of malignant neoplasms of the central nervous system (SIR, 1.71; 95% CI, 1.51–1.92). An association between agricultural exposure to herbicides or pesticides and tumors of the central nervous system has been reported (22). Pesatori et al. reported a tendency towards an increased risk of pituitary tumors in areas where the concentration of dioxin was higher (23). Although the exact mechanism is unclear, germline mutations in Ah receptor-interacting proteins may be associated with the incidence of these tumors, including familial pituitary adenomas (24).

Cancers of the head and neck, including malignant neoplasms of the eye, adnexa, lip, oral cavity, and pharynx also showed significantly elevated SIRs. Several studies have reported the possible mechanisms of carcinogenicity with regard to Ah receptor actions (25–27) as well as epidemiologic research results, such as the increased risk of head and neck cancers in Vietnam Era veterans (4, 28). The findings of this study were consistent with those of previous studies. One possible reason for the failure to find a significant association between exposure to TCDD or AO and cancers of the head and neck or central nervous system may be the small number of cases due to low incidence of cancers (4).

This study has several strengths. First, we used nationally representative data from the NHISS, which includes all Korean VW veterans and is the largest, most reliable, and objective data currently found in the world. The follow-up period was sufficiently long to observe the incidence of cancer, which may have been higher in older patients. Second, this was the first study to include female VW veterans. Although the number of female participants was much lower than that of male participants, investigating health risks in female veterans should be encouraged. Third, we found a significantly increased incidence of malignant neoplasms of the central nervous system, malignant neoplasms of the eye and adnexa, and other head and neck cancers. The risk of these cancers has not been conclusive, owing to the small number of cases. We believe that the elevated risk of these cancers observed in this study is actual, rather than coincidental.

However, this study has some limitations. It only demonstrated the risk of cancer in VW veterans who were alive during the baseline data collection of KOVECO in 2002. The VW ended in the mid-1970s, and early onset or high-mortality cancers occurring before the start year of the cohort study may not have been included because of lack of information from those periods in Korea. This is a type of left censoring cohort bias. However, the standards for the VW troop’s conscriptions were reasonably high at that time, and only the healthy population may have been dispatched to the VW. Thus, the current results may have clinical implications for chronic non-severe cancer epidemiology in VW veterans. Some Korean VW veterans may also have served in the Korean Civil War from 1950 to 1953 or in dioxin-contaminated areas in Korea from the late 1960s to the early 1970s. However, the number of those veterans is believed to be small. Most importantly, Korean veterans who served in the VW were exposed to AO, although the exposure levels may differ. Therefore, whether the veterans had served in both wars did not seem to affect the exposure profile and their cancer incidence rates significantly. Furthermore, our study relied on hospital visit information to assess cancer diagnoses, and did not obtain pathological confirmation. If patients had ICD codes for neoplasms for diagnosis before confirmation, they might not have been actual cancer cases. However, this error has no direction with regard to the VW veteran group or the non-VW veteran group in the age-sex-region matched cohort; hence, non-differential misclassification may have occurred. Consequently, the risk of cancer may instead have a forward-to-null relationship. We could not demonstrate cancer- related factors, such as screening results, familial history, or other medical conditions, due to the nature of the dataset. Further studies on cancer-related factors are warranted. Finally, other conditions, such as the areas where the veterans served, and environmental conditions, may have affected exposure and health outcomes. Unfortunately, we could not obtain information about other environmental conditions besides the variables of veterans dispatched to the VW included in the manuscript owing to security reasons and lack of data. However, herbicides were sprayed in almost all areas of Vietnam during the war, even outside the official operations field. Therefore, all Korean veterans who serve in the VW should be considered exposed to AO, although the exposure levels may differ. A comparison between different situations or times requires a cautious approach.

The risk of certain cancer types in Korean VW veterans was higher than expected. Although the toxicological effect of various chemicals used in the VW such as AO or dioxin could explain the large body of biological mechanisms, not all the oncologic effects on the VW veterans can be explained by current analysis. Further studies are warranted to investigate the various health determinants in veterans.

## Data availability statement

The datasets presented in this article are not readily available because they belong to the National Health Insurance System of Korea, and access requires certification. Requests to access the datasets should be directed to the corresponding author/s.

## Ethics statement

The studies involving human participants were reviewed and approved by the Institutional Review Board of Gachon University Gil Medical Center (IRB No. GCIRB2019-076). The patients/participants provided their written informed consent to participate in this study.

## Author contributions

S-KK and W-JC conceived and supervised the project. WL, SP, J-HY and W-JC conducted data curation, analysis, and prepared the data visualization. WL, J-HY and W-JC contributed to the validation of results and data. WL, SP, S-KK, SH, J-HY and W-JC conducted the study and drafted the manuscript. All authors contributed to the article and approved the submitted version.

## References

[1] Yi SW Ohrr H Hong JS Yi JJ. Agent orange exposure and prevalence of self-reported diseases in Korean Vietnam veterans. J Prev Med Public Health (2013) 46:213–25. doi: 10.3961/jpmph.2013.46.5.213 PMC3796647 24137524

[2] Stellman JM Stellman SD Christian R Weber T Tomasallo C. The extent and patterns of usage of agent orange and other herbicides in Vietnam. Nature (2003) 422:681–7. doi: 10.1038/nature01537 12700752

[3] National Academies of Sciences, Engineering, Medicine. Veterans and agent orange: Update 11. Washington, DC: The National Academies Press (2018). doi: 10.17226/25137 30629395

[4] Yi SW Ohrr H. Agent orange exposure and cancer incidence in Korean Vietnam veterans: a prospective cohort study. Cancer (2014) 120:3699–706. doi: 10.1002/cncr.28961 25103108

[5] Akhtar FZ Garabrant DH Ketchum NS Michalek JE. Cancer in US air force veterans of the Vietnam war. J Occup Environ Med (2004) 46:123–36. doi: 10.1097/01.jom.0000111603.84316.0f 14767215

[6] McBride D Cox B Broughton J Tong D. The mortality and cancer experience of new Zealand Vietnam war veterans: A cohort study. BMJ Open (2013) 3:e003379. doi: 10.1136/bmjopen-2013-003379 PMC3773658 24002985

[7] Lee W Kim UJ Ham S Choi WJ Lee S Yoon JH. Cohort profile: the Korean Vietnam war veterans’ health study cohort (KOVECO). Int J Environ Res Public Health (2022) 19:4211. doi: 10.3390/ijerph19074211 35409894 PMC8998788

[8] Lee W Kang MY Kim J Lim SS Yoon JH. Cancer risk in road transportation workers: A national representative cohort study with 600,000 person-years of follow-up. Sci Rep (2020) 10:11331. doi: 10.1038/s41598-020-68242-5 32647239 PMC7347601

[9] Lee W Kang MY Yoon JH. Cancer incidence among air transportation industry workers using the national cohort study of Korea. Int J Environ Res Public Health (2019) 16:2906. doi: 10.3390/ijerph16162906 31416127 PMC6727080

[10] IARC. Polychlorinated dibenzo-para-dioxins and polychlorinated dibenzofurans. IARC Monogr Eval Carcinog Risks Hum (1997) 69:1–631. doi: 10.1289/ehp.98106755 9379504 PMC5366851

[11] NTP. Report on carcinogen. 15th ed. research triangle park. NC: United States Department of Health and Human Services, Public Health Service (2021).

[12] Xu J Ye Y Huang F Chen H Wu H Huang J. Association between dioxin and cancer incidence and mortality: A meta-analysis. Sci Rep (2016) 6:38012. doi: 10.1038/srep38012 27897234 PMC5126552

[13] Villano CM Murphy KA Akintobi A White LA. 2,3,7,8-tetrachlorodibenzo-p-dioxin (TCDD) induces matrix metalloproteinase (MMP) expression and invasion in A2058 melanoma cells. Toxicol Appl Pharmacol (2006) 210:212–24. doi: 10.1016/j.taap.2005.05.001 15982688

[14] Seifert A Taubert H Hombach-Klonisch S Fischer B Navarrete Santos A. TCDD mediates inhibition of p53 and activation of ERalpha signaling in MCF-7 cells at moderate hypoxic conditions. Int J Oncol (2009) 35:417–24. 19578757

[15] Jain S Dolwick KM Schmidt JV Bradfield CA. Potent transactivation domains of the ah receptor and the ah receptor nuclear translocator map to their carboxyl termini. J Biol Chem (1994) 269:31518–24. doi: 10.1016/S0021-9258(18)31725-3 7989319

[16] Li W Donat S Döhr O Unfried K Abel J. Ah receptor in different tissues of C57BL/6J and DBA/2J mice: use of competitive polymerase chain reaction to measure ah-receptor mRNA expression. Arch Biochem Biophys (1994) 315:279–84. doi: 10.1006/abbi.1994.1501 7986069

[17] Whitelaw ML Gustafsson JA Poellinger L. Identification of transactivation and repression functions of the dioxin receptor and its basic helix-loop-helix/PAS partner factor arnt: inducible versus constitutive modes of regulation. Mol Cell Biol (1994) 14:8343–55. doi: 10.1128/mcb.14.12.8343-8355.1994 PMC359373 7969169

[18] Sorg O. AhR signalling and dioxin toxicity. Toxicol Lett (2014) 230:225–33. doi: 10.1016/j.toxlet.2013.10.039 24239782

[19] Saurat JH Kaya G Saxer-Sekulic N Pardo B Becker M Fontao L. The cutaneous lesions of dioxin exposure: Lessons from the poisoning of victor yushchenko. Toxicol Sci (2012) 125:310–7. doi: 10.1093/toxsci/kfr223 21998131

[20] Hayes JD Dinkova-Kostova AT McMahon M. Cross-talk between transcription factors AhR and Nrf2: lessons for cancer chemoprevention from dioxin. Toxicol Sci (2009) 111:199–201. doi: 10.1093/toxsci/kfp168 19628587

[21] Yi SW. Cancer incidence in Korean Vietnam veterans during 1992–2003: the Korean veterans health study. J Prev Med Public Health (2013) 46:309–18. doi: 10.3961/jpmph.2013.46.6.309 PMC3859852 24349652

[22] Piel C Pouchieu C Carles C Béziat B Boulanger M Bureau M. Agricultural exposures to carbamate herbicides and fungicides and central nervous system tumour incidence in the cohort AGRICAN. Environ Int (2019) 130:104876. doi: 10.1016/j.envint.2019.05.070 31344646

[23] Pesatori AC Baccarelli A Consonni D Lania A Beck-Peccoz P Bertazzi PA. Aryl hydrocarbon receptor-interacting protein and pituitary adenomas: A population-based study on subjects exposed to dioxin after the seveso, Italy, accident. Eur J Endocrinol (2008) 159:699–703. doi: 10.1530/EJE-08-0593 18787049

[24] Cazabat L Guillaud-Bataille M Bertherat J Raffin-Sanson ML. Mutations of the gene for the aryl hydrocarbon receptor-interacting protein in pituitary adenomas. Horm Res (2009) 71:132–41. doi: 10.1159/000197869 19188737

[25] John K Lahoti TS Wagner K Hughes JM Perdew GH. The ah receptor regulates growth factor expression in head and neck squamous cell carcinoma cell lines. Mol Carcinog (2014) 53:765–76. doi: 10.1002/mc.22032 PMC4388041 23625689

[26] DiNatale BC Schroeder JC Perdew GH. Ah receptor antagonism inhibits constitutive and cytokine inducible IL6 production in head and neck tumor cell lines. Mol Carcinog (2011) 50:173–83. doi: 10.1002/mc.20702 PMC3044777 21104991

[27] Kenison JE Wang Z Yang K Snyder M Quintana FJ Sherr DH. The aryl hydrocarbon receptor suppresses immunity to oral squamous cell carcinoma through immune checkpoint regulation. Proc Natl Acad Sci U.S.A. (2021) 118:1–12. doi: 10.1073/pnas.2012692118 PMC8126867 33941684

[28] Mowery A Conlin M Clayburgh D. Increased risk of head and neck cancer in agent orange exposed Vietnam era veterans. Oral Oncol (2020) 100:104483. doi: 10.1016/j.oraloncology.2019.104483 31810040

