# Supplementary material for: Cancer risk in Vietnam war veterans from the Korean Vietnam war veterans’ health study cohort
Source: Front Oncol. 2023 Jan 25;13:1048820. doi: 10.3389/fonc.2023.1048820 (PMC9905696; doi:10.3389/fonc.2023.1048820)
Supplement: Supplementary file 1 [file Table_1.docx]

**Supplementary table.** Age-specific hospital admissions status for neoplasms during the study period.

| Type of neoplasms | Vietnam-War Veterans | | | General population | | |
| --- | --- | --- | --- | --- | --- | --- |
|  | Hospital admissions cases | Person-year | Hospital admissions rate per 100,000 person-year | Hospital admissions cases | Person-year | Hospital admissions rate per 100,000 person-year |
| Malignant neoplasms of lip, oral cavity and pharynx |  |  |  |  |  |  |
| <56 | 6,146 | 1,384.1 | 444.0 | 23,204 | 5,318.6 | 436.3 |
| 56-60 | 14,309 | 2,657.1 | 538.5 | 63,736 | 11,877.7 | 536.6 |
| 61-65 | 3,234 | 467.7 | 691.5 | 9,597 | 1,511.3 | 635.0 |
| >65 | 1,773 | 215.8 | 821.7 | 4,321 | 526.2 | 821.2 |
| Malignant neoplasms of esophagus |  |  |  |  |  |  |
| <56 | 5,775 | 1,388.0 | 416.1 | 18,015 | 5,306.5 | 339.5 |
| 56-60 | 12,800 | 2,665.2 | 480.3 | 60,911 | 11,951.1 | 509.7 |
| 61-65 | 1,778 | 459.2 | 387.2 | 9,298 | 1,535.3 | 605.6 |
| >65 | 697 | 211.2 | 330.0 | 3,932 | 536.2 | 733.4 |
| Malignant neoplasms of stomach |  |  |  |  |  |  |
| <56 | 52,432 | 1,878.3 | 2,791.5 | 177,954 | 7,015.8 | 2,536.5 |
| 56-60 | 114,703 | 3,716.6 | 3,086.3 | 530,685 | 16,776.1 | 3,163.3 |
| 61-65 | 23,717 | 678.4 | 3,496.0 | 83,564 | 2,242.5 | 3,726.3 |
| >65 | 12,984 | 316.8 | 4,098.1 | 31,747 | 760.2 | 4,176.3 |
| Malignant neoplasms of colon |  |  |  |  |  |  |
| <56 | 20,792 | 1,546.4 | 1,344.5 | 77,307 | 5,961.2 | 1,296.8 |
| 56-60 | 50,099 | 3,056.0 | 1,639.4 | 234,594 | 13,863.8 | 1,692.1 |
| 61-65 | 12,195 | 564.7 | 2,159.5 | 36,503 | 1,826.7 | 1,998.3 |
| >65 | 7,654 | 271.2 | 2,822.3 | 15,310 | 638.2 | 2,399.0 |
| Malignant neoplasms of rectosigmoid junction, rectum, anus, and anal canal |  |  |  |  |  |  |
| <56 | 19,009 | 1,523.1 | 1,248.0 | 72,335 | 5,901.5 | 1,225.7 |
| 56-60 | 41,975 | 2,952.6 | 1,421.6 | 201,118 | 13,418.9 | 1,498.8 |
| 61-65 | 8,768 | 528.1 | 1,660.2 | 30,314 | 1,738.1 | 1,744.1 |
| >65 | 4,429 | 238.6 | 1,856.4 | 11,971 | 599.8 | 1,995.9 |
| Malignant neoplasms of liver and intrahepatic bile ducts |  |  |  |  |  |  |
| <56 | 34,080 | 1,687.2 | 2,019.9 | 127,438 | 6,470.1 | 1,969.6 |
| 56-60 | 69,037 | 3,248.9 | 2,124.9 | 315,109 | 14,527.2 | 2,169.1 |
| 61-65 | 11,525 | 558.6 | 2,063.3 | 41,202 | 1,844.3 | 2,234.0 |
| >65 | 4,961 | 248.5 | 1,996.2 | 13,176 | 613.1 | 2,149.2 |
| Malignant neoplasms of pancreas |  |  |  |  |  |  |
| <56 | 4,514 | 1,372.3 | 328.9 | 17,805 | 5,295.4 | 336.2 |
| 56-60 | 10,734.0 | 2,638.3 | 406.9 | 48,764.0 | 11,825.0 | 412.4 |
| 61-65 | 2,338.0 | 466.2 | 501.5 | 8,182.0 | 1,530.8 | 534.5 |
| >65 | 1,166 | 214.7 | 543.0 | 2,421 | 523.4 | 462.6 |
| Other malignant neoplasms of digestive organs |  |  |  |  |  |  |
| <56 | 5,975 | 1,387.4 | 430.7 | 21,643 | 5,337.7 | 405.5 |
| 56-60 | 11,704.0 | 2,643.6 | 442.7 | 65,627.0 | 12,012.4 | 546.3 |
| 61-65 | 3,066.0 | 472.0 | 649.6 | 10,819.0 | 1,559.1 | 693.9 |
| >65 | 1,550 | 219.1 | 707.4 | 4,188 | 536.8 | 780.1 |
| Malignant neoplasms of larynx |  |  |  |  |  |  |
| <56 | 5,199 | 1,379.8 | 376.8 | 14,353 | 5,247.1 | 273.5 |
| 56-60 | 12,740.0 | 2,652.4 | 480.3 | 47,334.0 | 11,788.9 | 401.5 |
| 61-65 | 2,785.0 | 468.8 | 594.1 | 8,061.0 | 1,519.8 | 530.4 |
| >65 | 1,334 | 215.5 | 618.9 | 2,885 | 525.3 | 549.2 |
| Malignant neoplasms of trachea, bronchus and lung |  |  |  |  |  |  |
| <56 | 38,030 | 1,788.1 | 2,126.9 | 109,225 | 6,388.4 | 1,709.7 |
| 56-60 | 92,795.0 | 3,609.7 | 2,570.7 | 358,688.0 | 15,378.1 | 2,332.5 |
| 61-65 | 20,084.0 | 663.0 | 3,029.2 | 64,996.0 | 2,113.8 | 3,074.8 |
| >65 | 10,328 | 303.6 | 3,401.9 | 24,282 | 708.8 | 3,425.7 |
| Other malignant neoplasms of respiratory and intrathoracic organs |  |  |  |  |  |  |
| <56 | 1,381 | 1,335.7 | 103.4 | 4,440 | 5,134.5 | 86.5 |
| 56-60 | 3,056.0 | 2,552.9 | 119.7 | 10,900.0 | 11,398.9 | 95.6 |
| 61-65 | 748.0 | 448.8 | 166.7 | 1,171.0 | 1,455.6 | 80.5 |
| >65 | 223 | 206.0 | 108.3 | 522 | 506.8 | 103.0 |
| Malignant neoplasms of bone and articular cartilage |  |  |  |  |  |  |
| <56 | 541 | 1,325.6 | 40.8 | 1,721 | 5,102.9 | 33.7 |
| 56-60 | 997.0 | 2,528.8 | 39.4 | 3,745.0 | 11,325.0 | 33.1 |
| 61-65 | 345.0 | 443.7 | 77.8 | 698.0 | 1,452.5 | 48.1 |
| >65 | 57 | 204.6 | 27.9 | 279 | 504.4 | 55.3 |
| Malignant melanomas of skin |  |  |  |  |  |  |
| <56 | 588 | 1,327.4 | 44.3 | 2,195 | 5,112.3 | 42.9 |
| 56-60 | 1,279 | 2,533.2 | 50.5 | 5,661 | 11,357.1 | 49.9 |
| 61-65 | 284 | 444.2 | 63.9 | 887 | 1,454.4 | 61.0 |
| >65 | 169 | 205.7 | 82.2 | 345 | 505.8 | 68.2 |
| Other malignant neoplasms of skin |  |  |  |  |  |  |
| <56 | 1,312 | 1,334.5 | 98.3 | 4,307 | 5,131.6 | 83.9 |
| 56-60 | 3,751 | 2,559.5 | 146.6 | 13,965 | 11,433.0 | 122.2 |
| 61-65 | 895 | 450.1 | 198.8 | 2,442 | 1,468.8 | 166.3 |
| >65 | 682 | 210.4 | 324.2 | 1,451 | 517.6 | 280.3 |
| Malignant neoplasms of mesothelial and soft tissue |  |  |  |  |  |  |
| <56 | 1,601 | 1,339.4 | 119.5 | 4,666 | 5,139.5 | 90.8 |
| 56-60 | 3,368 | 2,556.8 | 131.7 | 12,144 | 11,425.2 | 106.3 |
| 61-65 | 980 | 450.9 | 217.4 | 2,161 | 1,468.7 | 147.1 |
| >65 | 552 | 210.4 | 262.3 | 691 | 509.3 | 135.7 |
| Malignant neoplasms of breast |  |  |  |  |  |  |
| <56 | 398 | 1,325.0 | 30.0 | 1,502 | 5,106.8 | 29.4 |
| 56-60 | 711 | 2,528.1 | 28.1 | 2,704 | 11,326.2 | 23.9 |
| 61-65 | 330 | 445.1 | 74.1 | 739 | 1,452.5 | 50.9 |
| >65 | 127 | 205.7 | 61.8 | 199 | 505.1 | 39.4 |
| Malignant neoplasm of cervix uteri |  |  |  |  |  |  |
| <56 | 0 | 1,321.6 | 0.0 | 73 | 5,088.8 | 1.4 |
| 56-60 | 0 | 2,520.4 | 0.0 | 38 | 11,297.6 | 0.3 |
| 61-65 | 1 | 441.1 | 0.2 | 18 | 1,445.8 | 1.2 |
| >65 | 0 | 204.3 | 0.0 | 0 | 503.2 | 0.0 |
| Malignant neoplasms of other and unspecified parts of uterus |  |  |  |  |  |  |
| <56 | 0 | 1,321.6 | 0.0 | 3 | 5,088.4 | 0.1 |
| 56-60 | 2 | 2,520.4 | 0.1 | 37 | 11,297.7 | 0.3 |
| 61-65 | 0 | 441.1 | 0.0 | 0 | 1,445.7 | 0.0 |
| >65 | 0 | 204.3 | 0.0 | 5 | 503.2 | 1.0 |
| Other malignant neoplasms of female genital organs |  |  |  |  |  |  |
| <56 | 1 | 1,321.6 | 0.1 | 26 | 5,088.6 | 0.5 |
| 56-60 | 0 | 2,520.4 | 0.0 | 21 | 11,297.3 | 0.2 |
| 61-65 | 2 | 441.0 | 0.5 | 62 | 1,446.2 | 4.3 |
| >65 | 1 | 204.3 | 0.5 | 0 | 503.2 | 0.0 |
| Malignant neoplasms of prostate |  |  |  |  |  |  |
| <56 | 30,051 | 1,751.3 | 1,715.9 | 72,209 | 6091.5 | 1185.4 |
| 56-60 | 96,420 | 3,854.4 | 2,501.6 | 299,729 | 15317.5 | 1956.8 |
| 61-65 | 31,227 | 851.5 | 3,667.4 | 64,014 | 2260.0 | 2832.5 |
| >65 | 25,506 | 503.2 | 5,069.1 | 35,344 | 919.1 | 3845.7 |
| Other malignant neoplasms of male genital organs |  |  |  |  |  |  |
| <56 | 175 | 1,322.7 | 13.2 | 877 | 5095.0 | 17.2 |
| 56-60 | 512 | 2,524.0 | 20.3 | 2,061 | 11311.9 | 18.2 |
| 61-65 | 123 | 441.8 | 27.8 | 439 | 1449.6 | 30.3 |
| >65 | 84 | 205.0 | 41.0 | 189 | 504.5 | 37.5 |
| Malignant neoplasms of bladder |  |  |  |  |  |  |
| <56 | 10,607 | 1,448.8 | 732.1 | 33,088 | 5494.7 | 602.2 |
| 56-60 | 27,720 | 2,853.4 | 971.5 | 115,330 | 12669.5 | 910.3 |
| 61-65 | 7,020 | 523.3 | 1,341.6 | 20,862 | 1677.7 | 1243.5 |
| >65 | 5,629 | 263.5 | 2,136.2 | 11,176 | 620.4 | 1801.5 |
| Other malignant neoplasms of urinary tract |  |  |  |  |  |  |
| <56 | 7,555 | 1,413.2 | 534.6 | 23,332 | 5360.4 | 435.3 |
| 56-60 | 17,383 | 2,720.8 | 638.9 | 67,613 | 12070.2 | 560.2 |
| 61-65 | 3,366 | 478.4 | 703.6 | 9,473 | 1546.3 | 612.6 |
| >65 | 2,431 | 227.4 | 1,069.0 | 4,228 | 544.0 | 777.2 |
| Malignant neoplasms of eye and adnexa |  |  |  |  |  |  |
| <56 | 201 | 1,323.2 | 15.2 | 364 | 5090.7 | 7.2 |
| 56-60 | 429 | 2,524.5 | 17.0 | 1,092 | 11307.4 | 9.7 |
| 61-65 | 89 | 441.9 | 20.1 | 103 | 1446.2 | 7.1 |
| >65 | 27 | 204.3 | 13.2 | 72 | 503.5 | 14.3 |
| Malignant neoplasms of brain |  |  |  |  |  |  |
| <56 | 1,318 | 1,333.8 | 98.8 | 5,106 | 5136.8 | 99.4 |
| 56-60 | 3,041 | 2,547.5 | 119.4 | 13,244 | 11419.0 | 116.0 |
| 61-65 | 518 | 445.3 | 116.3 | 1,615 | 1460.2 | 110.6 |
| >65 | 267 | 206.1 | 129.6 | 869 | 510.6 | 170.2 |
| Malignant neoplasms of other parts of central nervous system |  |  |  |  |  |  |
| <56 | 247 | 1,323.3 | 18.7 | 761 | 5095.1 | 14.9 |
| 56-60 | 919 | 2,529.1 | 36.3 | 2,178 | 11314.3 | 19.3 |
| 61-65 | 313 | 444.4 | 70.4 | 401 | 1449.8 | 27.7 |
| >65 | 88 | 205.0 | 42.9 | 86 | 503.5 | 17.1 |
| Malignant neoplasms of other, ill-defined, secondary, unspecified, and multiple sites |  |  |  |  |  |  |
| <56 | 13,151 | 1,463.7 | 898.5 | 47,547 | 5605.0 | 848.3 |
| 56-60 | 26,276 | 2,790.5 | 941.6 | 117,533 | 12514.1 | 939.2 |
| 61-65 | 5,107 | 490.5 | 1,041.1 | 14,696 | 1574.6 | 933.3 |
| >65 | 2,891 | 229.4 | 1,260.0 | 4,523 | 538.9 | 839.2 |
| Hodgkin disease |  |  |  |  |  |  |
| <56 | 247 | 1,324.7 | 18.7 | 915 | 5096.6 | 18.0 |
| 56-60 | 657 | 2,526.7 | 26.0 | 2,225 | 11317.7 | 19.7 |
| 61-65 | 189 | 442.9 | 42.7 | 326 | 1449.3 | 22.5 |
| >65 | 130 | 205.4 | 63.3 | 42 | 503.2 | 8.4 |
| Non-Hodgkin lymphoma |  |  |  |  |  |  |
| <56 | 5,817 | 1,386.3 | 419.6 | 18,890 | 5299.3 | 356.5 |
| 56-60 | 13,624 | 2,678.1 | 508.7 | 50,332 | 11858.1 | 424.5 |
| 61-65 | 2,632 | 469.9 | 560.1 | 6,929 | 1516.0 | 457.1 |
| >65 | 1,855 | 220.2 | 842.2 | 2,548 | 526.2 | 484.2 |
| Leukemia |  |  |  |  |  |  |
| <56 | 3,417 | 1,364.4 | 250.4 | 11,953 | 5227.7 | 228.7 |
| 56-60 | 6,080 | 2,589.8 | 234.8 | 27,827 | 11614.0 | 239.6 |
| 61-65 | 1,353 | 455.4 | 297.1 | 3,614 | 1484.8 | 243.4 |
| >65 | 1,076 | 214.6 | 501.5 | 850 | 510.8 | 166.4 |
| Other malignant neoplasms of lymphoid, hematopoietic, and related tissue |  |  |  |  |  |  |
| <56 | 2,501 | 1,354.4 | 184.7 | 11,642 | 5236.7 | 222.3 |
| 56-60 | 8,386 | 2,624.6 | 319.5 | 30,480 | 11668.5 | 261.2 |
| 61-65 | 2,138 | 465.1 | 459.7 | 3,970 | 1492.6 | 266.0 |
| >65 | 1,031 | 214.5 | 480.8 | 1,566 | 517.7 | 302.5 |
|  | | | | | | |
